# Hormesis: induction by *Aloe vera* extracts and individual constituents

**DOI:** 10.1515/med-2026-1392

**Published:** 2026-05-11

**Authors:** Edward J. Calabrese, Peter Pressman, A. Wallace Hayes, Evgenios Agathokleous, Gaurav Dhawan, Rachna Kapoor, Vittorio Calabrese

**Affiliations:** Department of Environmental Health Sciences, Morrill I-N344, School of Public Health and Health Sciences, University of Massachusetts, Amherst, MA, USA; University of Maine, Orono, ME, USA; Center for Environmental Occupational Risk Analysis and Management, College of Public Health, University of South Florida, Tampa, FL, USA; School of Ecology and Applied Meteorology, Nanjing University of Information Science & Technology, Nanjing, China; Sri Guru Ram Das (SGRD), University of Health Sciences, Amritsar, India; Independent Consultant, Hartford, CT, USA; Saint Francis Hospital and Medical Center, Hartford, CT, USA; Department of Biomedical and Biotechnological Sciences, School of Medicine University of Catania, Catania, Italy

**Keywords:** hormesis, dose-response, biphasic, adaptogen, preconditioning

## Abstract

*Aloe vera* has an extensive history in traditional folk medicine. There is a robust scientific literature on *Aloe vera* extracts and several prominent chemical constituents, covering chemical characterization, biological/chemoprotective effects, and mechanisms. This paper provides the first integrative assessment of *Aloe vera*-induced hormetic dose responses. *Aloe vera* extract-induced hormetic responses acted as an immunostimulant in multiple fish species, enhancing development/growth and reducing adverse effects from environmental stressors. *Aloe vera* extract-induced hormetic effects are prominent in dermatological studies, improving keratinocyte- and fibroblast-related wound-healing processes. *Aloe vera* extracts induced hormetic-based neuroprotective endpoints for epileptic seizures, pain, in Alzheimer’s disease models, and for lifespan. This analysis includes the hormetic effects of specific chemical constituents of *Aloe vera* extracts (acemannan/aloin) on stem cells, cardiomyocytes, bone cells, and other cell types, and their mechanisms. These findings show that a complex range of *Aloe vera* extract mixtures/specific constituents induce hormetic dose responses. These findings also indicate that hormetic dose responses play a prominent role in mediating many biomedical and chemopreventive effects of *Aloe vera* extracts and their major chemical constituents. Recognition of these findings should assist in creating optimized hormetic-based study designs and improving biologically/mechanistically based interpretations of their dose-response findings with their clinical implications.

## Introduction

Over the past two decades, hormetic responses have been widely reported in the biomedical literature on numerous dietary (i.e., often called adaptogens) [1], 2] environmental [3], [4], [5], and medical substances as well as physical factors [6]. The published literature is sufficiently extensive with these findings being incorporated into a large hormetic database based on strict *a priori* criteria for study design, rigorous statistical criteria, and other features [7], [8], [9], [10]. This extensive literature has revealed that the hormetic dose response is a central feature of both growth (i.e., anabolic) and stress-induced adaptive (i.e., catabolic) processes [11] that govern life from before conception (i.e., oocyte maturation, sperm functionality), through each development/growth stage and adult life until death [2], 12], 13]. Not only is there extensive documentation that the hormetic concept dominates the biological effects of a vast range of endogenous metabolites and environmental toxicants, but also numerous dietary supplement adaptogens. The underlying mechanisms of many of these agents have been characterized, including their signaling pathways, interactions, and crosstalk [6], [14], [15], [16]. Despite its long history in the traditional folk medicine area, as well as extensive research concerning its biomedical effects and mechanisms, little effort has been directed towards clarifying the dose-response relationships of *Aloe vera* extracts and their principal constituents, especially in the low dose zone. Consequently, this paper provides the first comprehensive documentation and assessment of the occurrence of *Aloe vera* extracts and their constituents, focusing on hormetic dose responses and their clinical implications.

Although hormesis has been a widely cited biological concept, having received over 25,000 citations (i.e., using hormesis or hormetic) in the Web of Science data base in 2025 alone, published papers specifically linking the term hormesis and *Aloe vera* and/or its constituents have essentially not been cited in the major public scientific data bases such as Pub Med, Web of Science, Google Scholar and others. Thus, to undertake the present assessment of *Aloe vera* and its hormetic effects, novel, multifaceted, and iterative search strategies were required, such as the use of additional, broader descriptive terms and author and article follow-up strategies, to yield a more robust and representative set of citations for evaluation. Since *Aloe vera* extracts and some of its major constituents will be shown here to induce a wide range of hormetic dose responses, in the specific context of hormesis, a brief introduction/overview of the hormesis concept will now be provided.

## Hormesis overview

**Figure 1:**
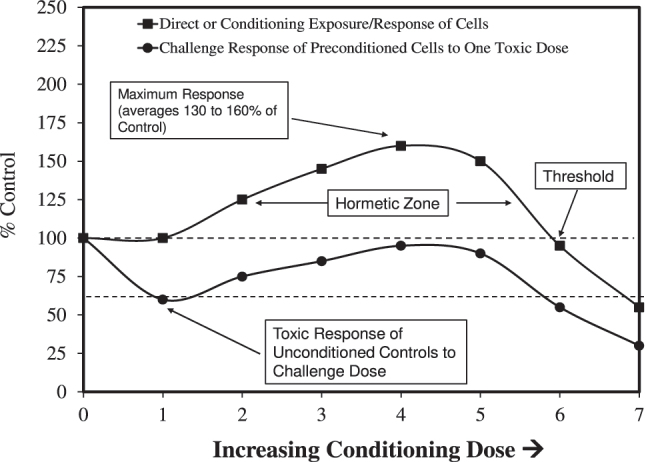
Dynamic features of the hormetic dose response for both direct and preconditioning experimental protocols [22].

Hormesis is a biphasic dose (or concentration) response, initially documented by Hugo Schulz in Greifswald, Germany, in the late 1880s [17], 18], showing that numerous toxic agents stimulated yeast metabolism at low concentrations [19], [20], [21]. Hormetic responses show a low-dose stimulation and a high-dose inhibition (Figure 1) [5], 7], 8], 19], 20], [22], [23], [24], [25]. This dose-response relationship shows specific quantitative features, with a maximum stimulation only modestly exceeding control group values by 30–60 %. The hormetic range of stimulatory doses is typically 10 to 20-fold below the toxicological or pharmacological threshold doses. Nonetheless, the hormetic dose-response range may display sizeable variability, often greater than 50-fold and, in some instances (i.e., several percent of reported examples), greater than 1,000-fold. The hormetic response can be induced by: 1) a direct exposure to a stress-inducing agent; 2) an hormetic conditioning dose [administered prior (preconditioning), concurrently, or following (post-conditioning) a toxic dose] [15], 16], 26]; or 3) a moderate overcompensation stimulation typically after a prior limited toxic exposure and/or a disruption in homeostasis [24], 26]. Of biological significance is that the hormetic dose response displays considerable generalizability [3], 4], [27], [28], [29], being independent of biological model (e.g., animals, microbes, plants), endpoint (e.g., growth, tissue repair, survival, reproduction), biological organization level (i.e., cell, organ, whole organism), *in vitro* or *in vivo* assessments, stress-inducing agent [20], 24], and underlying biological mechanism [6], 14]. Hormesis incorporates a capacity for assessing chemical mixtures, including additivity and synergism [24]. Low dose hormetic exposures to numerous chemical and physical agents display numerous adaptive/beneficial effects, including lifespan extension, enhanced development and growth, increased resistance to infection, reduced tumor incidence, and enhanced tolerance to toxic agents and radiation [20], 30].

From an evolutionary perspective, hormetic dose responses provide a highly conserved dose-response strategy for resource management, across numerous endpoints, cell types and species. The hormetic dose response defines the quantitative limits of biological plasticity along with the magnitude of stimulatory responses in constitutive/growth (anabolic) and adaptive (catabolic) processes [9], 11]. The hormetic dose response is therefore a fundamental feature of anabolic and catabolic metabolism.

The most credible explanation for why the hormesis concept was frequently missed or not taken seriously by biomedical researchers is that Schulz claimed to have discovered the explanatory principle of homeopathy [23], 31]. This action by Schulz quickly led to an academically hostile, aggressive, and prolonged rejection by the biomedical/clinical communities. The hormesis concept was also difficult to reliably detect as its modest maximum increases are only 30–60 % greater than the control response [8]. Until relatively recently, hormetic dose responses lacked adequate mechanistic explanations [9], 11]. However, each of these rather substantial historical, scientific, and biostatistical limitations has been largely addressed and overcome over the past two decades. The combination of these interacting significant factors led the biological, biomedical, and clinical communities to miss a fundamental evolutionary adaptive strategy with significant theoretical and practical implications for more than a century, during the critical period when therapeutic and environmental regulatory strategies were being developed worldwide.

## *Aloe vera* introduction

The present evaluation is dominated by biomedical evaluations intended to enhance human health and well-being, detailing hormetic effects across a wide range of biological systems and cell types. While many of the human-oriented studies have been conducted using *in vitro* systems, there is a substantial literature on the effects of *A. vera* extracts on the growth, development, and adaptive capacities of several fish species in *vivo* experimental settings. This paper will initially provide an assessment of fish research, followed by multi-system mammalian biomedical research.

## The effects of *A. vera* extracts on fish biology

### Tilapia

Tilapia is a common name for numerous species of cichlid fish. Cichlids share one important biological trait – the fusion of the lower pharyngeal bones into a single tooth-bearing structure. They display muscles that allow the upper and lower pharyngeal bones to be employed as a second set of jaws for processing food, permitting a division of labor between the “true jaws” (mandibles) and the “pharyngeal jaws”. Cichlids are efficient, typically highly specialized feeders that process a broad variety of food types, a factor that contributes to their diversity. Tilapia has become one of the most significant freshwater fish globally. Associated with the global production of tilapia has been its enhanced production and agricultural development resulting in greater biomass yield.

A component of the enhanced production of tilapia has been the use of immune-stimulation agents to activate and enhance nonspecific immune defense systems, thereby resisting infections and promoting general health indices. An herbal product known to affect immune stimulation and antiparasitic effects is *A. vera*, which contains a spectrum of chemopreventive agents, such as aloin, emodin, isobarbaloine, and acetylated mannose (acemannan) [32]. For example, acemannan enhances macrophage activity and modulates immune functioning by stimulating production and release of antibodies [33]. Within this context, a series of investigations have shown that *A. vera* extracts increased hematological parameters in tilapia [i.e., Nile (found in Nile River and tributaries) tilapia and GIFT (Genetically Improved Farm Tilapia) tilapia] in an hormetic manner. Initial studies were reported by Dotta et al. [34], 35] using Nile tilapia that were fed a combined extract of propolis and *Aloe* in equal proportions, although the method of *A. vera* extraction was not provided. These studies included three doses (0.5, 1.0, and 2.0 %) with exposure for two weeks. While only the thrombocyte hematological endpoints in the larger fingerlings were statistically significant [34], biphasic dose response patterns were a consistent observation across the multiple endpoints. These findings suggested the need to replicate the original findings and assess responses at lower concentrations. Calabrese and Baldwin [2] had earlier identified a series of age-dependent hormetic responses, showing differential susceptibilities and hormetic responses depending on dose and age.

**Figure 2:**
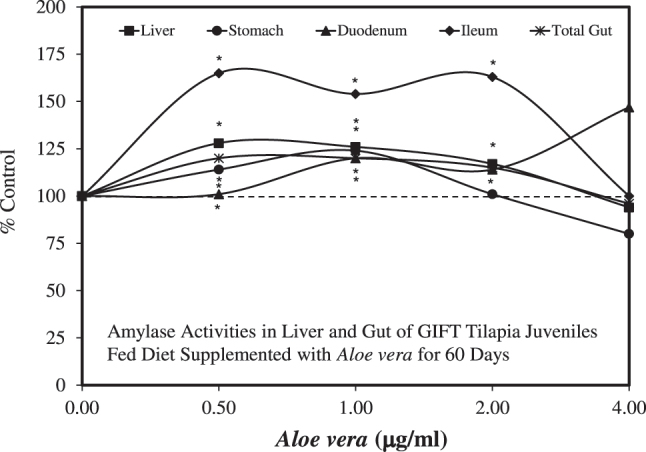
Amylase activities in liver and gut of GIFT tilapia juveniles fed diet supplemented with *Aloe vera* for 60 days (modified from [38]).

The findings of Dotta et al. [34], 35] were extended by Gabriel et al. [36], [37], [38] using a commercial powdered *A. vera* extract on GIFT tilapia with a focus on growth performance, as well as hematological and biochemical parameters. The Gabriel et al. [36], [37], [38] studies employed 75 fish per treatment group, followed over 60 days, using four doses (0.5, 1.0, 2.0, and 4.0 %). The *A. vera* treatments were associated with increases in weight gain/growth, red and white blood cell counts, and hematocrit at lower doses. As in Dotta et al. [35], the hormetic effects began at the 0.5 % concentration, the lowest tested. Hormetic endpoints were also extended to digestive enzymes (i.e., amylase activities) (Figure 2) for multiple tissues, as well as with high density lipoprotein (HDL) and hepatic glutathione peroxidase (GSHpx) levels. Similar findings were reported by Yunus et al. [39], who showed that *A. vera* extracts enhanced growth and red blood cell and white blood cell counts at 0.5 % in a 14-day study. The collective findings of these three independent research teams consistently showed that *Aloe* extracts enhanced the growth and hematological performance of the two tilapia models over the same dose range.

These collective findings reported a remarkably robust body of experimental data demonstrating the effects of dietary *A. vera* extracts on numerous, diverse, and fundamental health-based endpoints across several independent experimental commercial fish species studies. The studies were also striking in the consistency of the concentrations that induced hormetic dose responses. More recent complementary research by Mahato and Paudel [40] reported that *A. vera* supplementation at 0.5 % enhanced rainbow trout growth rate over 90 days. The growth in that study was not enhanced at the higher doses of 1.0 and 1.5 %. These findings provide a strong biomedical foundation for the application of such treatments within a commercial setting.

### Catfish

Commercial aquaculture has experienced a consistent annual growth of about 5 % over the past 25 years. Underlying this commercial success has been the integration of high-level production systems that achieve higher yields due to higher stocking densities. Such systems, however, generate stress in the stockfish, which can affect normal metabolism and meat quality while increasing disease susceptibility. As a result of these challenges, fish farmers have explored ways to maintain and enhance the health of their fish stocks. Initial strategies relied on synthetic pharmaceutical drugs. However, overtime, their use markedly declined due to changes in fish pathogen resistance, impairments in immune function, and unacceptable bioaccumulation. These changes have led to regulatory reforms in many countries requiring food products to be free of synthetic pharmaceutical drugs. These challenges created interest in natural product dietary supplements that may enhance fish health, growth and feed utilization along with an attractive public health quality profile. As a result, there has been considerable research interest in testing natural products across a broad range of commercial fish species for their potential health and food-quality benefits. Within this context, *A. vera* has generated considerable interest within the research community. The first publication of *A. vera* extracts on disease resistance in fish (i.e., rockfish) was published by Kim et al. [41], indicating that the consumption of *A. vera* extract showed an hormetic dose response for both serum lysozyme activity and chemiluminescence of head kidney leukocytes. Another *A. vera* extract product that induced an hormetic dose response was reported by Zanuzzo et al. [42] for the acute respiratory burst in pacu, an omnivorous South American freshwater fish. The authors indicated that their study and the earlier report by Kim et al. [41] were the only two to have assessed the potential role of *A. vera* in agriculture, even though it had been extensively used as a medicinal plant for a range of human applications. Emerging research by Kim et al. [41] and Zanuzzo et al. [42] suggested that *A. vera* extracts/constituents may have value in aquaculture, especially for enhancing nonspecific immune responses to prevent disease.

**Figure 3:**
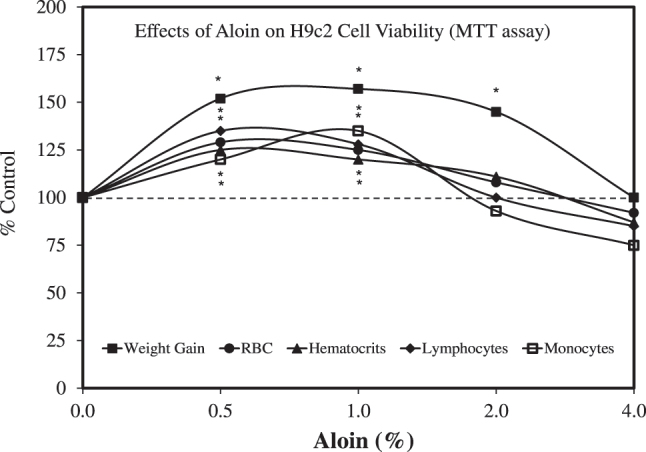
Effects of aloin on H9c2 cell viability (MTT assay) (modified from [45]).

These two studies showing dose-dependent increases in adaptive immune responses provided the foundation for a series of investigations by Gabriel et al. [36], [37], [38] with tilapia (see Tilapia section) which then led to his dissertation [43] and subsequent research on the effects of *A. vera* on the African catfish, an important aquacultural species in Namibia. The dissertation research was then published in several journal articles, with co-authors cited in the acknowledgements of his dissertation. The research by Gabriel et al. [44], 45] on the effects of *A. vera* used a commercial *A. vera* product, namely *A. vera* polysaccharide powder. Aloe polysaccharide contains a mixture of key components of *A. vera*, which have shown the capacity to induce hormetic effects individually (e.g., acemannan, aloin, aloe-emodin, and others). The protocol was similar to that reported in the tilapia research just described, involving the use of fingerlings and four doses (0.5, 1.0, 2.0, and 4.0 %) administered over 60 days. The results revealed hormetic dose responses for a range of growth-related variables (i.e., multiple growth parameters and weight gain parameters). Likewise, similar hormetic responses were reported for multiple hematological parameters (e.g., red blood cell count (RBC), hematocrit, hemoglobin, platelets, white blood cell count (WBC), monocytes, lymphocytes) (Figure 3) [45]. A similar hormetic response was observed when the fish were challenged with low pH for 3 days. A follow-up study was conducted with *A. vera* combined with dietary garlic, following the same general protocol and testing both agents separately [43]. The composite study also showed consistent hormetic effects across a broad spectrum of growth and hematological parameters. The hormetic maxima for the combined studies were similar to those of the individual-agent studies.

### Catla catla

In addition to research on tilapia and the African catfish, Yousaf et al. [46] assessed the effects of *A. vera* extract on the growth of fingerling *C. catla*, a freshwater fish with rapid growth and widely used in the aquaculture industry. The study followed the fish for 70 days, using five doses (1–5 %). As was the case with Tilapia and the African catfish species, the *A. vera* extract elicited a consistent, broad-spectrum hormetic effect on growth/weight gain, hematological parameters, antioxidant enzyme activities, and blood lysozyme activity and globulin levels. Lysosomal activity was measured because of its capacity to disrupt bacterial cell walls, thereby inhibiting bacterial growth.

### Carp

#### Immune stimulation

Diseases during the rearing of commercial fish species are a serious threat. Consequently, significant efforts have been made to address these challenges. Non-specific immunity is particularly important in protecting fish from pathogens. This has led to the use of immunostimulatory agents, especially since they tend to be low-cost and have greatly diminished toxicity risks. Within this context, Alishahi and Abdy [47] assessed the effects of *A. vera* extract from the inner gel, processed via an unspecified homogenization and filtration process, on the common carp, a significant commercial fish but one that has experienced high mortality due to infection. The Alishahi and Abdy [47] study assessed the effects of dietary *A. vera* extract on hemato-immunological parameters of carp at concentrations ranging from 0.1 to 1 % over 60 days. The results indicated an hormetic biphasic dose response relationship with respect to multiple growth-related endpoints as well as multiple immunological endpoints such as white blood cell counts and lysozyme activity levels. Lysozyme is an antimicrobial enzyme produced by animals that forms part of the innate immune system. Lysozyme is abundant in secretions, including tears, saliva, human milk, and mucus. It is also present in cytoplasmic granules of the macrophages and the polymorphonuclear neutrophils (PMNs). In addition, the mortality rate also showed an hormetic J-shaped dose-response relationship consistent with the changes observed with immunological and growth functions. These collective findings of the beneficial effects on growth rate, immune function, and mortality incidence led the authors to recommend the use of a 0.5 % dietary extract as an immune stimulant for the common carp.

#### Reduction of arsenic toxicity

The effects of toxic chemicals on fish have long been a significant environmental concern. This is particularly true because fish are typically at the top of the aquatic food chain and concentrate toxic metals. In an effort to protect fish from toxic metal contaminants, Batool et al. [48] suggested that dietary additives in fish feed can induce adaptive responses. Since *A. vera* extracts are well known for their antioxidant, anti-inflammatory, and immune stimulatory functions, Batool et al. [48] assessed their capacity to reduce arsenic toxicity in the white carp. The fish were fed a non-commercial, laboratory-processed *A. vera* extract prepared by a defined homogenization and filtering process of the *A. vera* inner gel for eight weeks at dietary levels of 1–5 %. The fish were also exposed to 1/6 and 1/3 of the sublethal arsenic concentration weekly. While these arsenic concentrations did not affect survival, the treatments decreased multiple growth parameters and intestinal enzyme activities, such as lipase. The *A. vera* extract reduced the toxicity of the arsenic following an hormetic dose response for all parameters measured, with the optimal dosage being 3 %. Based on these findings, the authors suggested that *A. vera* extract should be used as a supplement for white carp.

## Neuroscience

### Skin neuronal interactive effects

**Figure 4:**
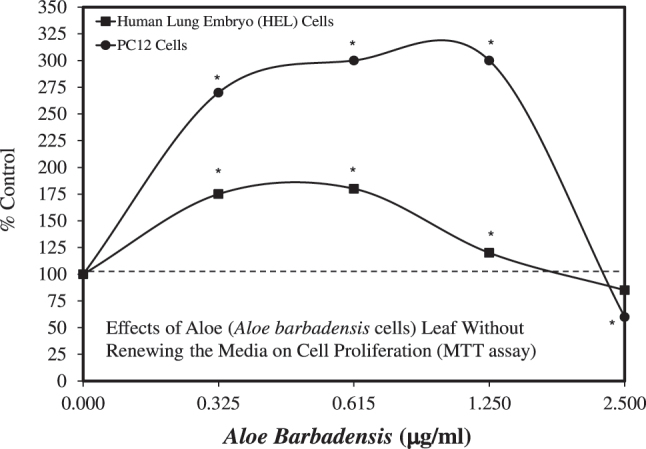
Effects of *Aloe* (*Aloe barbadensis* cells) leaf without renewing the media on cell proliferation (MTT assay) (modified from [49]).

In 1995, Bouthet et al. [49] became interested in whether *A. vera* applied for skin care would affect nerve cells adjacent to skin cells, such as fibroblasts and keratinocytes. As a result of that question, they assessed whether an *A. vera* extract could affect the cell viability of rat adrenal pheochromocytoma (i.e., a rare tumor that forms in the adrenal glands) PC12 cells and human embryonic lung cells (HEL). PC12 cells are not neurons themselves; they are a cell line derived from a rat pheochromocytoma, a tumor of the adrenal medulla. When exposed to nerve growth factor (NGF), PC12 cells may differentiate into neuron-like cells that exhibit a broad spectrum of properties characteristic of sympathetic neurons, making them a useful model for assessing neuronal function and differentiation. Using the MTT assay, they reported that the *A. vera* extract enhanced the proliferation of both cell types (PC12 and HEL) in an hormetic dose response fashion (Figure 4). While the PC12 cells showed greater stimulation than the HEL cells after 12 days, both cells showed an inhibitory response at the highest concentration tested. Since both cell types were stimulated at the three lowest concentrations, it is unknown how broad the stimulatory ranges are without further evaluation at lower concentrations. Given the consistency of the findings across the two cell types and the robust stimulation of cell proliferation, it is unclear why these encouraging findings have not yet been extended.

## Glufosinate and glutamate neurotoxicity

### Rat cortical cells

Glufosinate is a widely employed herbicide with neurotoxic potential, principally by altering synaptic regulation of glutamate. Given its neurotoxic potential, Yilmaz et al. [50] evaluated whether *A. vera* extracts could reduce glufosinate-induced toxicity in rat cortical cells using a post-conditioning protocol. Following 20 min of exposure to glufosinate, *A. vera* extract (50–1,600 ug/mL) was administered for 24 h. Using the MTT assay, the *A. vera* extract reversed the toxicity of glufosinate in this post-conditioning protocol before becoming toxic itself at higher doses. This pattern was also reported for total antioxidant enzymes. Using the same protocol, these authors reported similar biphasic hormetic responses to glutamate.

### HT-22 cells – derived from mouse hippocampal cells

It has been hypothesized that *A. vera* extract may protect neurons and related cells based on numerous reports of its antioxidant and anti-inflammatory properties, its capacity to prevent hepatotoxicity, and its capacity to augment wound healing and other therapeutic endpoints. Within this context, Jeon et al. [51] assessed the effects of *A. vera* extract on the viability of HT-22 cells challenged with glutamate in a preconditioning bioassay. In this study, the HT-22 cells were pretreated with the *A. vera* extract for 1 h prior to glutamate administration. The HT-22 cell line, derived from the mouse hippocampus, has been used to assess various aspects of glutamate-induced oxidative stress, leading to cell death via necrosis and apoptosis. In their study, Jeon et al. [51] reported that the *A. vera* extract prevented glutamate-induced cell death. The mechanism of neuroprotection was related to its capacity to reduce elevated intracellular calcium levels induced by glutamate treatment. The preconditioning study was limited to three doses, thereby precluding an adequate dose-response appraisal.

## Epileptic seizures

In 2005, Inoue et al. [52] first reported that complex N-095, containing 10 mg of aloe wood, significantly reduced the occurrence of toxic seizures induced by phenylenetetrazol (PTZ) and significantly prolonged survival time in a mouse model. Follow-up studies have shown that an aqueous extract from *A. vera* leaves significantly increased the latency to the onset of clonic convulsions while decreasing seizure duration in a rat model [53]. Subsequent research by Wang et al. [54] reported that a metabolite of *A. vera*, aloesone, induced a J-shaped dose response for PTZ-induced seizures, showing an hormetic dose response relationship, reducing seizure frequency by approximately 50 % at the optimal dose. Mechanistic follow-up studies associated the aloesone-induced protection with its capacity to modulate the phosphorylation of the non-receptor tyrosine kinase (SRC), which had been identified as the target of aloesone based on network pharmacology and molecular docking studies. The biphasic effects on SRC phosphorylation were closely associated with aloesone’s anti-seizure effects. These studies show that aloesone prevented PTZ-induced seizures by inducing activation of non-receptor cytoplasmic tyrosine kinase (c-SRC), suggesting that it may serve as an anti-seizure agent. It should be noted that numerous anti-seizure drugs that have been tested in the PTZ seizure model have been shown to act via hormetic dose-response relationships similar to that reported herein with aloesone [20].

## Pain

**Figure 5:**
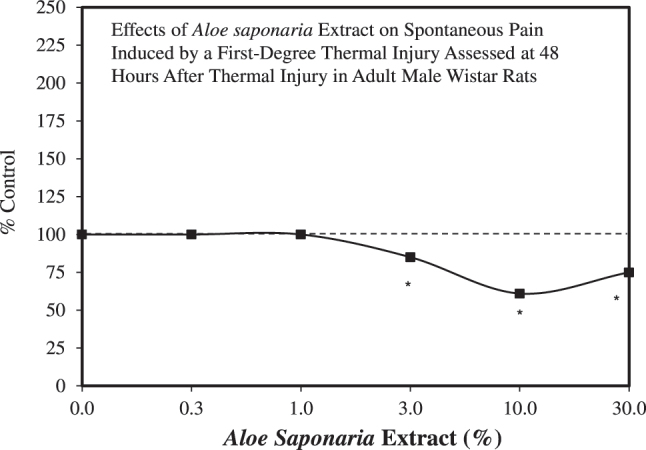
Effects of *Aloe saponaria* extract on spontaneous pain induced by a first-degree thermal injury assessed at 48 h after thermal injury in adult male Wistar rats (modified from [61]).

Thermal injury is a serious medical concern, often resulting in substantial inflammation and tissue damage, making the affected area susceptible to infection. In folk medicine, topical *A. vera* gel has been commonly used to treat burn injuries and enhance wound healing [55]. The folk medicine experience has been supported by a series of preclinical investigations addressing the anti-inflammatory healing properties of *Aloe* extracts [56], [57], [58]. Furthermore, there is clinical evidence that *A. vera* is more effective than silver sulfadiazine, a standard treatment for burn injuries [59], 60]. In this context, Silva et al. [61] assessed pain response parameters and the anti-inflammatory effects of *Aloe saponaria* extract on thermal first-degree injury in adult male Wistar rats. The injury was induced by immersion in water at 70° centigrade for 5–10 s, depending on the experiment. The injured animals were topically treated with *Aloe* extract (0.03–30 % concentration range) soon after thermal stress, then once per day thereafter for up to 6 days. The *Aloe* extract displayed a J-shaped dose response with the optimal concentration being 10 % (Figure 5). This concentration was then selected for follow-up experiments in a series of studies involving second-degree burns, paw edema, leukocyte infiltration, and other parameters. The protective effects of the *A. vera* extract were demonstrated in subsequent experiments, supporting its potential clinical applications.

## Dermatological effects

### Fibroblasts

There is a long but scattered history of components of *A. vera* reported to stimulate proliferation of a wide range of cell types [62], [63], [64], [65], [66], [67]. In addition, the *A. vera* components enhanced keratinocyte migration to wound sites [62]. Despite this broad range of experiments, none had assessed the role of *A. vera* on skin fibroblasts. This research framework led to a breakthrough paper by Abdullah et al. [68] on the effects of *A. vera* on skin fibroblasts. In that study, human type 2 diabetic (insulin-dependent) and non-diabetic skin fibroblast cell lines from the forearms of individuals aged 35–45 years were assessed by cell count after 3 days of *A. vera* treatment (0.6–10.0 %). The proliferation rate of *A. vera*-treated diabetic fibroblasts (without FGF-2 added) increased by about 15–20 % in the 1.2–2.5 % concentration range, then decreased at higher concentrations, showing a typical hormetic concentration response. In contrast, the proliferation of non-diabetic fibroblasts was not stimulated by the *A. vera* extract treatment by itself but only in the presence of FGF-2. Of interest is that the *A. vera* treatment also enhanced gap junction intercellular communication in an hormetic manner, closely corresponding to the diabetic fibroblast hormetic effects on cell proliferation. In general, this indicated that the *A. vera* treatment was effective in altering the diabetic and non-diabetic fibroblasts, depending on the presence of FGF-2 and the involvement of gap junction intercellular communication.

**Figure 6:**
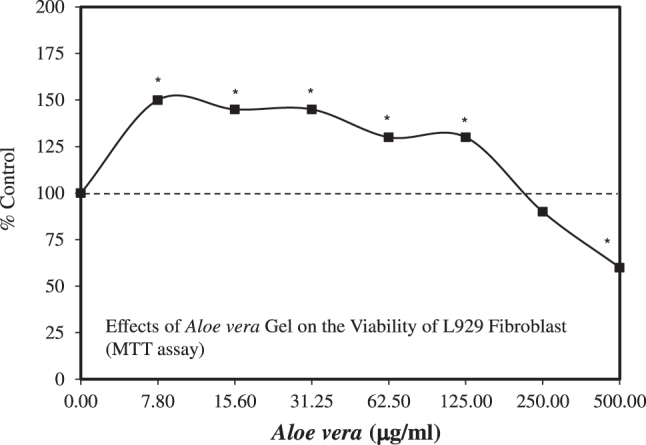
Effects of *Aloe vera* gel on the viability of L929 fibroblasts (MTT assay) (modified from [69]).

Despite these promising findings, research on the role of *A. vera* and its principal constituents in fibroblasts and keratinocytes of normal and diabetic humans, along with its gap junction mechanisms, was not extended. What followed has been research using different mouse fibroblast lines, including the L929 (Figure 6) [69], [70], [71], NIH 3T3 cells [72], and the mouse skin fibroblast cell line c147 [73]. While each of these investigations revealed hormetic biphasic dose response relationships for cell viability/proliferation and cell migration, each incorporated unique specific experimental protocols, precluding the capacity to compare these investigations directly. Of importance is that the *A. vera* extract procedures varied widely across the studies, with no confirmation of the chemical composition of the extracts used.

In three cases, the papers assessed *A. vera* extracts, while the fourth [73] assessed *A. vera* gel. The endpoints were measured over 24, 48 and 72 h, with three studies reporting values for 24 h, four for 48 h and three for 72 h. Only the study of Negahdari et al. [73] reported values for the three time points. Each study employed the MTT assay, except that Iosageanu et al. [71] used the neutral red assay.

**Figure 7:**
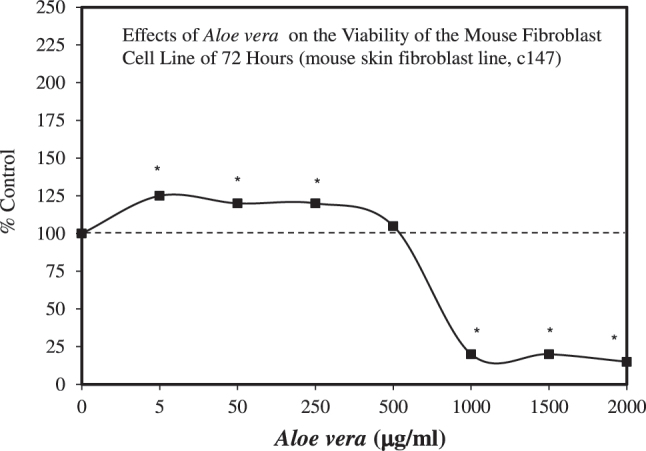
Effects of *Aloe vera* on the viability of the mouse fibroblast cell line of 72 h (mouse skin fibroblast line, c147) (modified from [73]).

This composite type series of variable study design research strategies yielded several general conclusions. The data indicated that *A. vera* extracts and gel were consistent in enhancing cell viability/cell proliferation at 24 h. The dose response closely conformed with the quantitative features of the hormetic dose response. However, there was some inter-study variability in responses over longer durations. The studies differed widely in doses assessed. Some studies assessed responses over a relatively low narrow range [e.g., Manoj et al. [70]: 10–75 ug/mL; Iosogeanu et al. [71]: 0.1–3.0 mg/mL]; and others for a broader range [e.g., Rizqi and Fitriawan [72]: 5–500 ug/mL; Negahdari et al. [73]: 5–2,000 ug/mL] (Figure 7)]. In general, these studies used a reduced number of doses, derived from the optimal stimulatory zone for cell viability, to test cell migration responses, precluding the assessment of the quantitative features of possible hormetic effects. The exception was Rizqi and Fitriawan [72], who evaluated the entire dose range, showing an hormetic dose response for both cell proliferation and cell migration. Mechanism pathway analyses were not addressed in these papers despite their relatively recent publication dates.

## Keratinocytes

The effects of *A. vera* extracts on keratinocytes have generated a broad range of research interests. These included their capacity to enhance skin elasticity [74], to prevent hair loss [75], and to protect against toxic threats such as UV [74] and ionizing radiation [76]. In addition, *Aloe* extracts were tested for their capacity to enhance keratinocyte cell viability [75], cell proliferation and differentiation [77], 78], and showed evidence of hormetic dose responses [78]. Similar findings were reported for ginseng, green tea, and shitake [75]. A follow-up preconditioning study by He et al. [79] indicated that aloin induced a protective effect on HaCaT cells in an hormetic manner when stressed by UV.

These findings indicate that *A. vera* can protect the skin against UV-induced damage. Likewise, the authors suggested that *A. vera* has the potential to be applied to prevent hair loss [75]. While there are several dozen patents on *A. vera* enhancing hair growth, the peer-reviewed literature is limited, suggesting that follow-up research related to the Kim et al. [75] study is warranted.

## Bone

### Aloe polysaccharides

Non-digestible bioactive polysaccharides have become of considerable biomedical interest. Particular focus has been on their capacity to prevent osteoporosis and other bone disease conditions. For example, the polysaccharide from *Polygonatum sibiricum* enhanced osteoblast differentiation, reducing the risk of osteoporosis [80]. Mechanistic studies indicate that protective effects involve activation of the ERK/GSK-3β/β-catenin pathway. *A. vera* extract also contains a diverse and substantial range of bioactive substances, including aloe polysaccharides (AP). Such APs are recognized for their powerful antioxidant, anti-inflammatory, immunoregulatory, antiviral, and antibacterial functions. Building on the earlier Peng et al. [80] study with *P. sibiricum* polysaccharide, Yao et al. [81] assessed the capacity of AP to promote osteogenic and adipogenic differentiation in adipose-derived stem cells (ADSCs). ADSCs have multidirectional differentiation capacities, being able to be transformed into adipocytes, osteoblasts, and chondrocytes. Within this context, Yao et al. [81] reported that AP enhanced the proliferation (CCK-8 assay) of ADSC’s as derived from the bilateral inguinal fat of young Sprague Dawley rats (gender not given). Using five concentrations of AP, the AP induced an hormetic-biphasic dose response after 72 h. Having established an optimized hormetic stimulatory concentration for cell proliferation, Yao et al. [81] then extended these findings by demonstrating that AP can induce adipogenic differentiation within a hormetic stimulatory framework. Follow-up mechanistic evaluation established that AP enhanced osteogenesis of ADSCs via the BMP2/SMAD signaling pathway(s) while concurrently inhibiting lipogenic differentiation.

### *Aloe vera* ethanolic extracts

In 2023, Blanco et al. [82] reported that multiple agents in *Aloe* extracts, such as anthraquinones, polyphenols, phenolic acids, coumarins, and glycosides, enhanced osteoblast activity *in vitro* and *in vivo* [83], [84], [85]. Several other investigators have also shown that *A. vera* extracts positively enhance osteoblast function [86], [87], [88]. As a result of these investigations, Blanco et al. [82] evaluated the effects of *Aloe arborescens* extracts on primary osteoblasts from neonatal rat calvaria (upper part of the skull). Based on studies with ethanolic extracts, they reported that the *Aloe* extract enhanced cell viability (neutral red stain) and cell proliferation (crystal violet stain) in an hormetic manner at 72 h. Similar findings were reported for cell migration at 8 and 24 h. Cell differentiation experiments evaluating mineralization at 17, 21, and 25 days also displayed hormetic dose response relationships. The authors noted that agents, such as acemannan polysaccharides, did not affect their stimulatory responses, as they would not have been extractable with ethanol.

## Kidney cells

*A. vera* extracts (AVE) have generated considerable interest with their potential antiviral effects. In laboratory studies assessing the antiviral effects of *A. vera* extracts, experiments typically used green monkey kidney cells, called Vero cells. The name Vero is an aberration of *Verda Reno*, which means green kidney in Esperanto. A significant feature of Vero cells is that they are interferon-deficient, meaning they lack the capacity to produce antiviral interferon proteins when infected with a virus. This biological feature makes this cell type useful in virology, facilitating the production of high virus titers without the ability to mount a defense. Thus, the use of Vero cells enhances the efficiency of generating and isolating large quantities of virus for study and vaccine production. Within this context, Kambizi et al. [89] assessed the capacity of AVE to affect the production of herpes simplex virus type 1 when grown in a Vero cell culture. Of interest to the present paper is that the AVE enhanced the viability of the Vero cells, using the MTT assay, showing an hormetic dose response. The hormetic response is practically important in the present case, since it can reduce virus production at high doses but increase it when the growth curve is optimal, that is, at the hormetic optima. It should be noted that Vero cells were used in the production of the Salk polio virus, with the addition of ethanol enhancing virus production in an hormetic manner [90].

## Reproductive cells

### Ovarian follicles

Calabrese et al. [12] identified approximately 70 agents that act in an hormetic fashion to enhance a broad spectrum of oocyte maturation, blastocyte and embryo development features. These hormetic actions were observed with chemically diverse substances across a broad spectrum of biological models. Within this context, Azevedo et al. [91] reported that an *A. vera* extract enhanced the growth of normal bovine secondary follicles over six days. Follicles that contained highly organized granulosa cells along with an intact oocyte and basement membranes were designated as normal. The determination of follicular growth was based on measuring follicular diameter. The enhanced follicle growth was associated with a similar hormetic dose response with calcein staining, which was employed to visualize and assess cell viability in live cell imaging, revealing biphasic treatment effects as a biomarker of cell viability and membrane integrity. Other hormetic biphasic concentration responses were reported with superoxide dismutase (SOD), peroxidreoxin-6, GSHpx, and other antioxidant enzymes. Likewise, the antrum cavity of the follicle showed an hormetic response with the maximum response reported at 2.5 uM. Overall, follicle health was maximized at 2.5 uM, with the onset of differential stress and toxicities at progressively higher concentrations. Further research at concentrations below 2.5 uM may be warranted to determine whether optimal hormetic responses occur at lower concentrations.

## Semen

Preservation of semen is a significant issue in animal husbandry. A large literature exists on this issue, with many agents having been evaluated for their cryopreservation potential, including many plant-derived antioxidants [92]. For example, detailed evaluations of these agents have consistently shown that numerous flavonoids can act as semen preserving agents, doing so within an hormetic biphasic dose-response manner. Within this context, it is not surprising that *A. vera* extracts have also been evaluated. For example, Pimpa et al. [93] assessed the effect of *A. vera* gel on rooster sperm quality and fertility. Using 30- to 40-week-old roosters, the effects of *Aloe* gel at 7 concentrations (0.25–20 %) were evaluated after varying storage durations. Following 72 h of storage, the *A. vera* gel treatment enhanced sperm viability and motility in an hormetic-like biphasic dose response manner. Consistant findings were reported with malondialdehyde (MDA), showing a J-shaped dose response.

These findings have potential practical significance, as rooster semen can be stored at 5 °C for 24 h. The addition of the *Aloe* treatment extended the 5 °C-based protection for 72 h. However, the study was not designed to separate the effects of *A. vera* from those of 5 °C temperature with precision. Nonetheless, the report of Pimpa et al. [93] is complementary to the research of Agbaye et al. [94] with semen from the red Sokoto buck, which reported the protection of semen after thawing following cryoprotection, again showing an hormetic dose response.

## Intestine

The effects of *A. vera* and related products on intestinal health have been reported with a strong focus on viral-induced diarrhea [95]. Particular interest has been directed to how to effectively treat viral porcine epidemic diarrhea (PED), now a worldwide disease that was first reported in 1978. Combating this virus-induced diarrhea has involved various strategies, such as different types of vaccines, but with only limited success. This has led to the need to broaden strategic considerations, including the potential use of natural products that have antiviral properties. Among the candidate agents are *Aloe* extracts, which are inhibitory against a broad range of specific viral agents. Within this framework, Xu et al. [96] reported the effects of *A. vera* extract on PED viral inhibition using IPEC-J2 cells and subsequent studies in piglets. In their assessment of the cytotoxicity of *A. vera* extract, Xu et al. [96] exposed IPEC-J2 cells to the extract for up to 48 h and evaluated cell viability/proliferation using the CCK-8 assay. The *A. vera* extract displayed an hormetic effect at both the 24- and 48-h time points with the optimal concentration being 16 mg/mL. The dose-response findings were striking since an exposure only twice the optimal beneficial concentration resulted in over 90 % loss of cell viability. These findings led to the suggestion that future experiments be conducted at concentrations below 16 mg/mL. However, switching to a lower concentration to avoid toxicity also significantly reduces the magnitude of the hormetic stimulation. Antiviral assays revealed that *A. vera* induced a dose-dependent decrease in virus production, with none observed at 16 mg/mL. Studies with weaned piglets by Qiao et al. [95] reported that *A. vera* polysaccharide enhanced growth in an hormetic dose response manner that was related to its capacity to improve intestinal tract health, reducing the occurrence of diarrhea.

An earlier study by Picchietti et al. [97] also reported that *A. vera* extract significantly affected piscine SAF-1 cells, a fibroblast-like culture derived from the marine fish gilthead seabream (*Sparus aurata*). This cell type has been found to be an effective model for research on fish pathology, particularly involving viruses and bacteria, as well as its capacity to display adaptive responses. Picchietti et al. [97] reported that the *A. vera* extract induced an hormetic dose response for ATP production, which is a manifestation of an adaptive response. The low-dose stimulatory effect of ATP (1.2 mg/mL) was subsequently reported to act as a potent immune stimulant in LPS- or poly (I: C)- activated SAF-1 cells, showing synergistic effects on the production of key biomarkers (e.g., IL-1β, TGF-β, COX-2, TNFα). It is important to note that it is unusual for investigators to identify an optimal hormetic dose and then to evaluate its capacity to interact, as shown here with LPS.

### Stem cells

**Figure 8:**
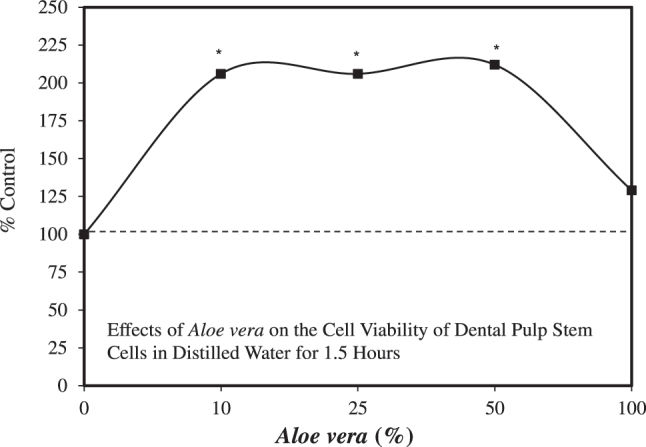
Effects of *Aloe vera* on the cell viability of dental pulp stem cells in distilled water for 1.5 h (modified from [99]).

Dental pulp stem cells (DPSCs) have been shown to have a significant role in tissue regeneration [98]. The DPSCs can differentiate into a broad range of cell types, including odontoblasts, osteoblasts, chondrocytes, adipocytes, neuronal cells, amongst others. Of practical interest is that teeth may need to be stored, typically following traumatic injuries. Immediate transfer of removed teeth is needed to enhance tooth cell viability. When prolonged time is required for tooth transportation, Hanks balanced salt solution (HBSS) is typically an efficient/acceptable storage medium. HBSS helps maintain pH and osmotic balance. The HBSS will also provide water and valuable inorganic ions. Despite HBSS’s central role in sustaining tooth viability, Sholehvar et al. [99] assessed whether *A. vera* gel could serve as a possible replacement for HBSS as a transportation medium (Figure 8). The *A. vera* treatment displayed a marked hormetic biphasic dose response value for DPSCs viability over multiple time periods. The *A. vera*-treated cells displayed significantly greater survival than those treated with HBSS after 6 h. The *A. vera* treatment also displayed an hormetic response at 1.5, 3, and 6 h, within the same optimal concentration range, outperforming the HBSS.

## Lifespan

**Figure 9:**
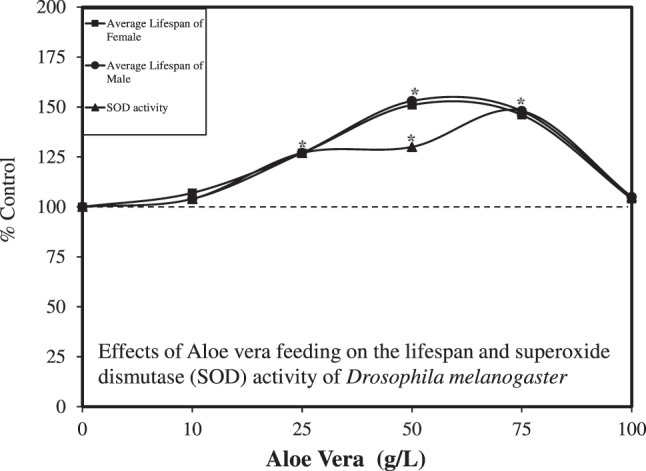
Effects of *Aloe vera* feeding on the lifespan and superoxide dismutase (SOD) activity of *Drosophila melanogaster* (modified from [100]).

In 2011, Deng [100] assessed the effect of *Aloe* juice from the leaves with the leaf peel removed on the lifespan of *Drosophila melanogaster*. Exposure began within 8 h of emergence from the pupal stage to the adult stage. In their study, the *Aloe* material was added to the culture medium as a food source. Five concentrations of the *Aloe* juice were used, ranging from 10 to 100 g/L solutions. The *Aloe* treatment displayed an hormetic effect on lifespan, showing a biphasic dose response for male and female flies. Male and female flies showed similar quantitative features of the dose response, with both displaying statistically significant increases at 25, 50, and 75 g/L, with the optimal range for the males and females being 50–75 g/L. The maximum increase was approximately 50 % for both the average and maximum lifespan. Of particular interest is that the 50 % increase at 75 g/L returned to control values as the dose increased to 100 g/L. Thus, there was a quick drop in the enhanced response as the dose exceeded 75 g/L (Figure 9). Data collected on tissue SOD levels showed a similar hormetic dose response pattern, with the optimal dose being 75 g/L. The authors suggested that aloe polysaccharides likely helped mediate the life-extending responses, probably by enhancing immune function and repair activities. Despite the striking and consistent effects of *Aloe* leaf juice on lifespan, these findings were not extended by other *Drosophila* researchers.

A life span study considering the effects of *A. vera* gel on *Caenorhabditis elegans* was reported by Berk [101]. This investigation also included an assessment of the effects of the *A. vera* gel on fertilization and developmental endpoints such as body size. The *C*. *elegans* were raised on a nematode growth medium containing concentrations of *A. vera* gel ranging from 0.312 to 5 mg/mL. The *A. vera* gel treatment increased the number of eggs in the fertilization assay, showing an hormetic dose response with the maximum stimulation being approximately 30 %. The body size (i.e., length essay) also showed an hormetic dose response with the optimal response occurring at 2.5 mg/mL, showing a stimulation of approximately 25 %. The lifespan study also showed an hormetic dose response with the maximum occurring at the 2.5 mg/mL concentration, with the stimulation being reported as 18.6 %. The findings of Berk [101] indicate that *A. vera* gel was consistent in inducing hormetic dose responses across multiple reproductive, developmental, and aging-related endpoints, with optimal responses at the same concentration of 2.5 mg/mL. Mechanistic follow-up studies indicated that the enhanced longevity was associated with increased expression of various RNAs involved in the mTOR pathway.

## Acemannan

### Macrophage

Acemannan, a polysaccharide from *A. vera* gel, has generated widespread interest for its potential biomedical applications over nearly five decades. Acemannan has been reported to enhance wound healing [102], 103], reticulo endothelial functioning [104], 105], cell mediated immunity [106], and hematopoietic proliferation and differentiation [107], 108]. All such functions have been regarded as secondary to the stimulation of the macrophage-monocyte cell lineage, leading to enhanced cytokine secretion. Within this context, a water-soluble acemannan called CARN 750 was developed and acts largely by activating monocyte macrophages, thereby affecting the release of a host of cytokines [e.g., interleukin 1 (IL-1), IL-6, interferon (IFN), GM-CSF, and TNF]. Experiments with myelo-suppressed female C57BL/6 mice (via high dose of radioactivity) showed that CARN 750 biphasically enhanced the formation of white blood cells, platelets, spleen cells, lymphocytes, PMNs, and monocytes [109]. The biphasic dose responses conformed closely to the quantitative features of the hormetic dose response [109].

### Stem cells – dental pulp stem cells

Despite active research on acemannan regarding macrophage activation, subsequent research has focused on specific stem cell populations, including dental pulp stem cells (DPSCs) and bone marrow stem cells (BMSCs). Interest in the effects of acemannan on DPSCs arose initially when Jittapiromsak et al. [110] reported that acemannan enhanced oral wound healing by stimulating gingival fibroblast proliferation, collagen synthesis, and vascular endothelial growth factor expression.

These effects on soft tissue raised the question of whether acemannan could enhance the proliferation of hard tissues such as dentin. Experiments with primary DPSCs showed that acemannan biphasically enhanced proliferation, with a similar dose-response pattern to that reported for differentiation endpoints, such as APL on days 3 and 9, in male Sprague-Dawley rats. Acemannan, therefore, has the capacity to enhance cell proliferation and differentiation in the same biological model. The authors concluded that acemannan enhanced dentin formation by enhancing primary DPSC proliferation, differentiation, extracellular matrix formation, and mineralization.

**Figure 10:**
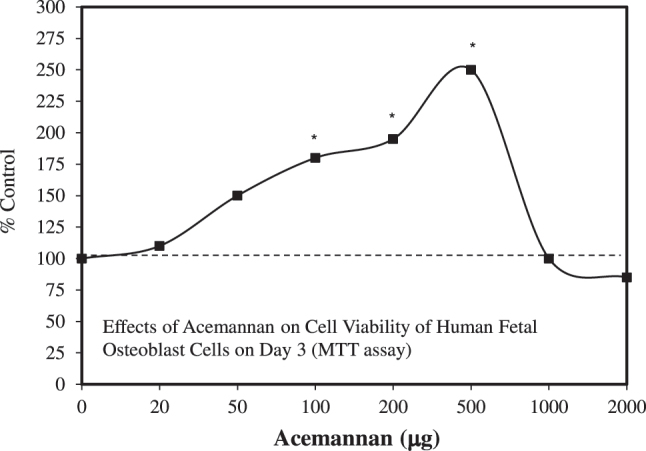
Effects of acemannan on cell viability of human fetal osteoblast cells on day 3 (MTT assay) (modified from [111]).

These findings led to follow-up research by Banerjee et al. [111], which showed that acemannan from hydroxyapatite-coated titanium enhanced the viability of human fetal cells on days 1 and 3 using the MTT assay (Figure 10). *In vivo* experiments showed that the release of acemannan resulted in nearly 60 % new bone formation during the early stages of regrowth, with greater mineralization by five weeks following implantation. Only one dose was tested *in vivo,* precluding an assessment of hormesis.

### Bone marrow mesenchymal stromal cells (BMSCs)

**Figure 11:**
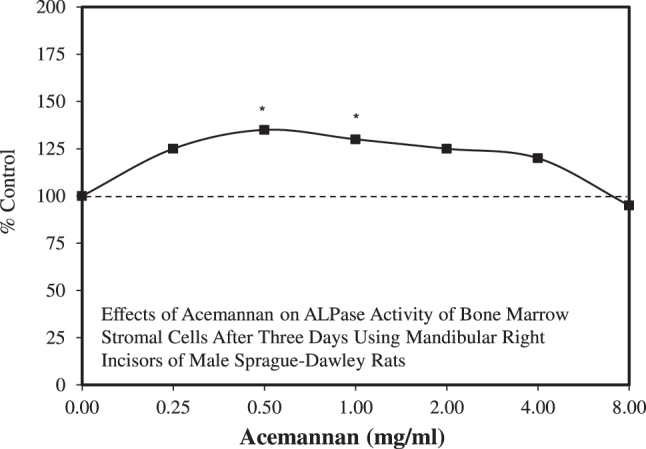
Effects of acemannan on ALPase activity of bone marrow stromal cells after three days using mandibular right incisors of male Sprague-Dawley rats (modified from [112]).

Boonyagul et al. [112] reported that acemannan enhanced BMSC proliferation and VEGF expression in a dose-dependent manner. Over the same dose range, the acemannan enhanced cell proliferation and ALP activity at day 3 (Figure 11). In contrast to the dose-dependent responses for cell proliferation and VEGF, the effect on ALP was biphasic, showing features of a hormetic dose response. A similar pattern was reported for BMP-2. However, in contrast to ALPase, which is an early stage of osteoblast differentiation, later differentiation stages involving OPN, BSP, and osteocalcin showed dose-dependent increases. The different response patterns were addressed to some extent in a speculative manner. For example, they suggested that different signaling pathways may regulate the different patterns. They noted that pre-osteoblast cells exhibit different responses to TGF-β at different concentrations [113].

## Aloin

### Heart

**Figure 12:**
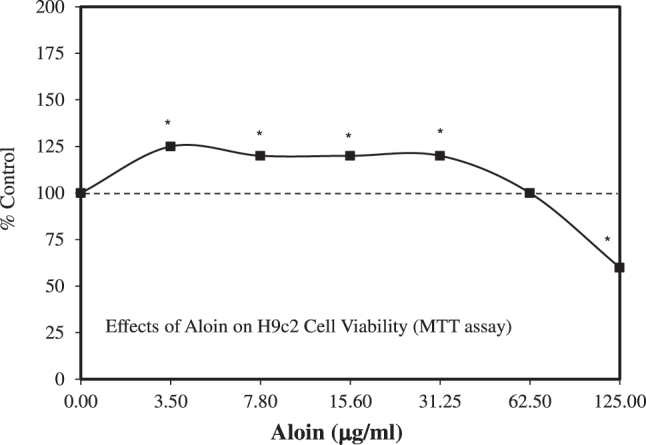
Effects of aloin on H9c2 cell viability (MTT assay) (modified from [115]).

Aloin is an anthraquinone glycoside found in *A. vera* extract. Interest has been directed toward better understanding its specific effects and how it may affect the overall impact of *A. vera* extracts on biological systems. Since aloin has been shown to effectively diminish oxidative stress and inflammatory cytokine concentrations as well as to inhibit ischemia/reperfusion-induced damage and inflammatory responses in cardiomyocytes via NrF-2 activation [114], Syed et al. [115] extended this research to assess how aloin may affect cardiac hypertrophy and myocardial fibrosis in a rat model as well as via the use of H9c2 cardiomyocytes (Figure 12). The H9c2 cell model study showed that aloin enhanced cell viability in an hormetic fashion using the MTT assay. In the rat study, aloin reduced left ventricular stiffness in a hormetic manner. The findings suggest that aloin offers promise for therapeutic application to prevent cardio hypertrophy and fibrosis.

**Figure 13:**
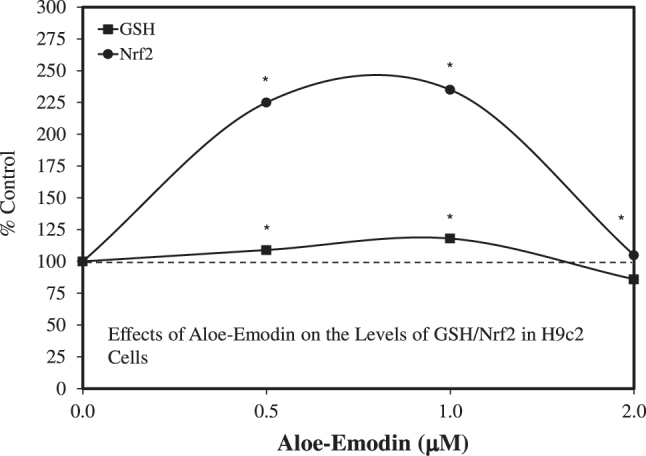
Effects of aloe-emodin on the levels of GSH/Nrf2 in H9c2 cells (modified from [117]).

Supporting this perspective was the report by Yang et al. [116], which showed that aloin treatment enhanced the function and maturation of human cardiomyocytes derived from human pluripotent stem cells within a hormetic-based preconditioning protocol. Likewise, He et al. [117] reported that aloe-emodin alleviated doxorubicin-induced cardiotoxicity by inhibiting ferroptosis through upregulation of GSH and NrF2 in a hormetic manner (Figure 13). While immediate implications suggest a unique strategy for producing greater numbers of adult-like cardiomyocytes for application in regenerative medicine, it also further extends the generality of aloin’s cardioprotective effects and their potential for widespread clinical and public health applications.

### Bone

In a manner similar to that reported for *Aloe* extracts, aloin has also been shown to enhance the viability of MC3T3-E1 cells using the MTT assay in an 11-concentration study spanning a 1000-fold concentration range [118]. Not only did aloin enhance cell viability, but it also enhanced cell differentiation at several of the concentrations. Mechanistic investigations using pathway inhibitors of p38 MAPK, SAPK/JNK, and Wnt-5a blocked the cell-differentiation-stimulatory effects. These findings confirmed that aloin’s effects are mediated through the MAPK and WNT/BMP signaling pathways.

## Aloe-emodin

### Cancer

Aloe-emodin displays anticancer effects on multiple tumor cell types, including neuroectodermal tumors, lung squamous cell carcinomas, and hematomas [119], [120], [121]. Aloe-emodin inhibited tumor cell proliferation by blocking S-phase progression, generating ROS, and preventing the activation of extracellular signal-regulated kinases. This finding led Lin et al. [122] to assess the effects of aloe-emodin on human colon cancer cells. In this case, the aloe-emodin enhanced cell viability of the DLD-1 human colon carcinoma cell line by about 20 % over a nearly tenfold concentration range. The concentration range was high, being in the low millimolar range. However, even at the lowest concentration employed (i.e., 0.04 mM–40 μM), there was no decrease in stimulatory activity. Of further interest is that aloe-emodin protected glioma cells from TNFα-induced toxicity, showing a striking hormetic biphasic dose response [123]. However, in the absence of the TNFα-induced toxicity, the aloe-emodin failed to show clear evidence of the biphasic dose response with toxicity at higher doses. According to Harhaji et al. [123], these findings showed that aloe-emodin prevented inflammation, thereby enhancing survival at low concentrations. These findings of Lin et al. [122] and Harhaji et al. [123] are consistent with the report of Calabrese [124] on the hormetic effects of low doses of large numbers of anti-tumor agents. The aloe-emodin-induced protection against TNFα-induced toxicity was associated with the inhibition of ERK activation, but not with TNFα-induced ROS production or p38 MAPK activation in tumor cells.

## Discussion

*A. vera* and other *Aloe* species have supported the preparation of extract mixtures that induce a wide range of chemo-preventive and growth-related responses (Table 1). These effects have been reported in both *in vivo* and *in vitro* experimental systems. Furthermore, the effects have been reported for complex mixture extracts and with specific identified chemotherapeutic agents such as acemannan, aloe-emodin, aloin, aloesone, β-sitosterol, and other agents, with each showing hormetic dose responses. The *A. vera* extracts also contain varying amounts of additional hormetic acting agents such as polyamines [30], 130], and multiple phenolic acids and flavonoids [131], [132], [133] such as rutin [134], quercetin [135], kaempferol [22], chlorogenic acid [136], caffeic acid [137], ferulic acid [138], naringin [92], and luteolin [139]. *Aloe* extract mixtures, therefore, contain multiple agents that have been shown to induce hormetic dose responses in numerous biological systems for a broad range of endpoints. Thus, the *A. vera* extract represents a complex mixture of many agents that have been well studied for their capacity to induce hormetic effects. However, how these agents interact in biological systems to induce hormetic effects is largely unknown. Nonetheless, when these individual agents have been studied in mechanistic experiments, specific pathways and their interactions have been identified, as noted in the above-cited papers for each specific *A. vera* extract constituent. The effects of the *A. vera* extract mixtures have been shown to activate both catabolic and anabolic processes and their underlying mechanistic pathways that mediate these responses [11].

**Table 1:** Summary of experimental studies of *Aloe vera* extracts induced-hormetic effects.

Author	Model	Endpoints	Number of doses	Maximum stimulation, %	Stimulatory doses	Stimulatory range
*Aloe vera* extracts						
Dotta et al. [35]	Tilapia	RBC	3	119	1	NA
		WBC	3	106	1	NA
		Thrombocytes	3	172	3	4-fold
		Lymphocytes	1	108	1	NA
		Neutrophils	1	131	1	NA
Gabriel et al. [36]	Tilapia	HDL	4	132	3	4-fold
		GSHpx	4	133	3	4-fold
Gabriel et al. [37]	Tilapia	RBC	4	133	1	NA
		Hemoglobin	4	140	1	NA
Gabriel et al. [38]	Tilapia	Amylase				
		Liver	4	128	3	NA
		Stomach	4	124	2	NA
		Duodenum	4	120	2	NA
		Ileum	4	165	3	NA
		Total gut	4	120	3	NA
Yousaf et al. [46]	*Catla*	Weight gain	5	195	5	5-fold
		Peroxidase	5	136	4	4-fold
		Catalase	5	146	4	4-fold
		SOD	5	182	5	5-fold
		Hemoglobiin	5	116	3	3-fold
		Platelets	5	117	3	3-fold
Gabriel [43]	African catfish	Final weight	4	118	1	NA
		Protein efficiency	4	125	1	NA
		Hematocrit	4	120	3	4-fold
Kim et al. [41]	Rockfish	Lysozyme activity	2	131	1	NA
		Leukocyte chemilumin.	2	147	1	NA
Gabriel et al. [45]	African catfish	Weight gain	4	152	3	4-fold
		Food efficiency	4	161	3	4-fold
Blanco et al. [82]	Neonatal rat calvarial cells	Cell viability	3	125	3	5-fold
		Cell prolif.	3	130	2	2.5-fold
		Mineralization	3	180	2	2.5-fold
Kim et al. [75]	HUVEC	Capillary formation	3	133	2	10-fold
Silva et al. [61]	Male Wistar rat	Pain	5	61-J shaped	3	10-fold
Qian et al. [76]	HaCaT	CCk-8 assay	5	129	2	2-fold
Zanuzzo et al. [42]	Pacu fish	Respiratory burst	4	125	3	5-fold
Deng [100]	Fruit fly male	Lifespan	5	153	3	3-Fold
Deng [100]	Fruit fly female	Lifepan	5	151	3	3-Fold
Kim et al. [75]	HaCaT	WST-1	5	111	3	4-fold

**Acemannan**						

Jittapiromsak et al. [110]	Dental pulp stem cells	Cell prolif.	5	205	5	8-fold
		ALP activity	5	150	2	2-fold
Jattanacheawhintit et al. [125]	Human gingival fibroblasts	Collagen production	7	180	6	32-fold
Sahawat et al. [126]	Human cementoblasts	Total collagen	5	150	4	8-fold
		VEGF	5	162	4	8-fold
		Mineral deposition	5	135	5	16-fold
Boonyagul et al. [112]	Bone marrow stem cells	ALPase	6	140	4	8-fold
Godoy et al. [127]	Female Sprague Dawley rat	Bone surface	4	135	4	2-fold
		Bone volume	4	145	4	4-fold
Banerjee et al. [111]	Human fetal osteoblasts	MTT	7	240	5	20-fold

**Aloin**						

Yang et al. [116]	Human pluripotent stem cells	Cardiomyocyte index	5	119	1	NA
Syed et al. [115]	H9c2 cells	MTT	6	125	4	9-fold
Pengjam et al. [118]	MC3T3-EI cells	MTT	11	115	1	NA
Luo et al. [128]	RAW 264.7	MTT	5	124	3	2-fold
Wang et al. [54]	BMSC	CCK-8	7	110	1	NA
Dinc et al. [129]	Human dental pulp	WST-1	5	253	5	20-fold
Harhaji et al. [123]	glioma	Cell prolif crystal violet	6	144	5	8-fold

The hormetic dose responses occur whether the effects of individual mixture components in *Aloe* extracts are assessed or if the extract mixture is evaluated. Within this context, the quantitative features of the dose responses are limited to the hormetic maximum of 30–60 % which is constrained by the limits of biological plasticity [9]. Even when hormetic synergies have been reported, the maximum stimulatory response is still constrained by the limits of biological plasticity [19]. The concept of hormetic synergy differs from that of toxicological synergy in that hormetic synergies are limited to 30–60 %, whereas there are no apparent plasticity constraints on toxicological synergies.

Of particular significance in the present assessment has been the highly consistent *Aloe*-induced hormetic dose responses within a dietary framework across multiple fish species (e.g., tilapia, catfish, carp). Studies enhancing growth and resistance to various types of chemical, biological, and physical stresses have been reported. These findings have been highly consistent across multiple species regarding dosing strategies, suggesting that *A. vera* may become a significant dietary additive in commercial fish and shellfish farming. These findings have been consistently robust and extend across a broad range of biologically notable health-related endpoints, leading these researchers to recommend their use as a dietary supplement for commercial applications in aquaculture.

The present findings indicate that *A. vera* extract comprises numerous constituents that exhibit chemopreventive activity across multiple biological systems in a hormetic dose-responsive manner, enhancing growth and development while also affecting a broad spectrum of adaptive responses, protecting against toxic stressors of normal metabolism, aging processes, and environmental stressors.

## Conclusions

This paper has demonstrated that *A. vera* extracts and its major chemical constituents induce hormetic effects in a wide range of biological models, affecting a broad spectrum of organ systems and cell types. These hormetic effects have been reported in both whole animal and *in vitro* experimental systems. Many of the investigations demonstrated that *A. vera* extracts and chemical constituent treatments are effective in preventing damage from a range of stressor agents. In addition, the *A. vera* extracts and chemical constituents are also effective in enhancing growth and development via growth related anabolic processes as well as in extending longevity. Detailed, pathway-based mechanistic investigations are needed to clarify the mechanisms by which these hormetic effects are mediated.
